# Clinical decision tools are needed to identify HIV-positive patients at high risk for poor outcomes after initiation of antiretroviral therapy

**DOI:** 10.1371/journal.pmed.1002278

**Published:** 2017-04-18

**Authors:** Margaret L. McNairy, Elaine J. Abrams, Miriam Rabkin, Wafaa M. El-Sadr

**Affiliations:** 1 ICAP, Columbia University, New York, New York, United States of America; 2 Department of Medicine, Cornell Medical College, New York, New York, United States of America; 3 Department of Epidemiology, Mailman School of Public Health, Columbia University, New York, New York, United States of America

## Abstract

Margaret McNairy and colleagues highlight the need for clinical decision tools to help identify HIV patients who would benefit from tailored services to avoid poor outcomes such as death and loss to follow-up.

**Summary points**

- Current interest in differentiated models for HIV care has largely focused on the needs of stable patients who are doing well on antiretroviral therapy. However, tailored services are also needed for patients at the other end of the spectrum, those at high risk for poor outcomes such as death and loss to follow-up.
- Clinical decision tools such as risk scores are needed to identify patients at high risk for poor outcomes and to provide individualized risk assessment.
- Risk scores need to be simple to use, utilize routinely collected variables, be accurate in predicting risk, and be generalizable across contexts.

## Introduction

Over the past decade, the scale-up of HIV programs in resource-limited settings has been remarkable, with over 17 million persons initiating lifesaving antiretroviral therapy (ART) by the end of 2015 [1]. However, in order to reduce HIV-related morbidity and mortality and decrease the number of new infections, it is critical to double the number of HIV-positive patients on treatment by 2020 [2]. It is equally important for health outcomes among children and adults on ART to be optimized [2,3]. To achieve these two goals—i.e., a massive increase in the number of patients on ART as well as an enhancement in the quality of care—the global health community has recognized the need for tailored HIV services to meet the unique needs of different patient groups, often referred to as differentiated models of service delivery [4].

The World Health Organization (WHO) recommends differentiating patients into four groups: those presenting with asymptomatic HIV infection at ART initiation, those initiating ART with advanced HIV disease, those who are already on ART but are unstable on treatment, and lastly those stable on ART with high adherence and a favorable clinical response [5]. However, to date, the focus has largely been on establishing differentiated service models for stable patients with the goal of maintaining adherence and retention, enhancing patient satisfaction, and increasing efficiencies in HIV programs, given the reality of overcrowded clinics that strain the capacity of the limited health workforce. Patient-managed community ART groups, one example of a differentiated service delivery model for stable patients, require fewer clinical assessments at health facilities. This in turn decompresses health facilities, relieves the workload of health care workers, and enables the patients to avoid transport costs, long wait times, and time lost from family or work [6]. Eligibility criteria for these groups and similar less intensive models of care are often based on prior performance—i.e., evidence of 6–12 months of excellent clinic attendance with high adherence to treatment and, if available, evidence of viral suppression [7,8]. Reported outcomes among such groups are impressive, with over 95% of patients retained at 1 year [7,9].

## The need for a priori identification of patients at high risk for poor outcomes

Patients at high risk for poor outcomes, on the other hand, also require a service model that is tailored to their unique needs. The first group of such patients consists of those at high risk for early death after ART initiation. Adults with advanced HIV disease have been noted to have mortality rates up to 5-fold higher in the first 6 months after treatment initiation compared with after 1 year [10,11]. Mortality rates also remain high in the first year of treatment among more recent cohorts with less advanced disease [12]. In a study of 19 cohorts with advanced disease from sub-Saharan Africa, the mortality rate in the first 4 months after ART initiation was 19.1 deaths per 100 person years (PY), decreasing to 1.3 deaths/100 PY beyond 1 year of follow-up [11]. Similarly, in a multicountry study of over 37,000 HIV-infected children, the mortality rate in the first 6 months was 9.1 deaths/100 PY versus 4.5 deaths/100 PY at 24 months [13]. This high early mortality is particularly evident among patients with HIV-related tuberculosis, cryptococcal meningitis, or malnutrition [14–16]. For children, poor growth, advanced disease, and young age are also predictive of early death [13,16].

The second group of patients at high risk for poor outcomes consists of those who default early from care. Rates of loss to follow-up among adults and children are highest in the first year after enrollment in care or initiation of treatment, with up to one-third of adult patients noted to be lost to follow-up within the first 6 months [12,17,18]. Outcomes among those who default from care are poor. In a meta-analysis of 28 studies conducted in sub-Saharan African countries reporting outcomes among patients who were categorized as lost to follow-up with unknown vital status and were traced in the community, the unreported mortality was 30% (95% CI 21.1%–38.9%) [19]. Moreover, patients enrolled in care but who have yet to initiate ART who experience more than a 12-month gap in their follow-up were found to re-present to clinics with more advanced disease, which could have been prevented with more consistent engagement in care [20]. Similarly, tracing studies among children who have been lost to follow-up found that 16% of HIV-positive children and 29% of children with unknown HIV status had died [21].

The evidence of poor outcomes among the two groups of patients described above demonstrates that it is critically important to identify, a priori, these patients at the time of ART initiation in order to prevent their loss to follow-up and to improve their clinical outcomes. Risk scores and clinical decision tools have been used widely in clinical medicine to simplify and standardize the identification of individuals at highest risk for a specific condition or health outcome. For example, risk scores are used to predict patients at high risk for cardiovascular disease [22] and pneumonia-related mortality [23] and to determine the need for hospitalization among patients presenting with syncope [24]. In the context of HIV, risk scores have been developed based on data from United States and European cohorts to assess short-term disease progression in HIV-positive patients on ART, 1-year mortality, and viral suppression, but none have been developed based on information from HIV-positive patients in low- and middle-income countries [25–27].

## Risk factors for poor outcomes in HIV are known, but individual prediction tools are lacking

While multiple studies have identified individual risk factors associated with early death and loss to follow-up among HIV-positive patients in resource-limited settings, work is needed to translate univariable and multivariable models into validated and easy-to-use risk scores to predict an individual’s risk of death and loss to follow-up after ART initiation [11,28–31]. In contrast to the use of CD4+ count or WHO HIV disease staging to identify high-risk patients for disease progression, a composite risk score may provide more specific information on the magnitude of risk for each patient by integrating additional variables such as weight, history, or the presence of a specific opportunistic infection and other parameters. Risk scores also may identify patients who may not have advanced HIV disease but are at high risk of poor outcomes because of a combination of socioeconomic or demographic variables such as limited income, un- or underemployment, and a fragile social support network. For example, while most providers are likely to intuit that a patient with a CD4+ count < 50 cells/mm^3^ or with concurrent tuberculosis is at high risk for early mortality even after initiation of ART, there is more ambiguity in determining the risk of early mortality for a patient with a CD4+ count of 250 cells/mm^3^ who lives alone and is unemployed.

Similar to the assessment of risk of early mortality, it is also critical to identify those at high risk for loss to follow-up at the time of ART initiation. Among adults, factors associated with loss to follow-up include male sex, adolescence and young adulthood, low income, and low educational attainment [12,32]. For children, factors include age (<5 years as well as adolescence), stigma from peers, family and community members, transport to clinic, and lack of disclosure of HIV infection to the child [13,21,33]. There are other potential variables that may be associated with loss to follow-up to increase the precision of a risk score such as distance to clinic, prior clinic attendance, family support, and social stressors that could be incorporated in deriving precise risk scores for loss to follow-up [34].

Ideally, risk scores for both early mortality and for loss to follow-up should be based on routinely collected clinical, laboratory, and demographic information in order to enable their utilization across contexts. Fortunately, many HIV programs routinely collect common patient variables such as age, sex, marital status, pregnancy status, weight, CD4+ count, WHO disease stage, history, and current tuberculosis (TB) status among other parameters. Other potential variables that can be easily obtained include education level, income, other comorbid diseases, the presence of other household members with HIV, disclosure status, the presence of depression, and health facility characteristics such as the level of the facility (e.g., hospital versus health center) and the distance of the health facility from the patient’s home.

A useful risk score must be distinguished by its potential for easy incorporation into clinical practice, be reliable when applied by diverse cadres of health care providers, and be accurate in predicting risks across settings [35]. It is important to avoid the complexity of some existing risk scores that involve lengthy calculations and subtle clinical judgment, which is subject to variation by provider. Once a risk score is proposed for identifying patients at high risk for poor outcomes, it must then be externally validated to determine if its accuracy is preserved when applied to a new population or setting and also over time. It is also important for the proposed risk score to undergo formal impact analysis to evaluate if the score or prediction rule influences physician behavior and patient outcomes, a step that is neglected for many proposed prediction rules [36,37]. While a risk score may have excellent accuracy, it is only useful if it will be used by providers to change or direct their decisions. And finally, a fourth phase of research is to evaluate if the rule has been actually implemented in practice [37].

## Differentiated HIV service delivery for high-risk patients

While the development of simple risk scores to identify patients at high risk for poor outcomes could be of substantial value, further efforts are needed to define interventions and care models to minimize the risk of these poor outcomes. Interventions to mitigate early mortality after ART initiation for patients with advanced HIV disease may include hospitalization of severely ill patients, providing prophylaxis or pre-emptive therapy for opportunistic infections, and/or more intensive clinical visit schedules. The WHO guidelines recommend for such patients the rapid initiation of ART, screening and prompt treatment for coexisting TB or cryptococcus, and provision of isoniazid preventive therapy, if indicated, as well as intensive follow-up for patients with CD4+ count < 200 cells/mm^3^ or WHO stage III/IV disease [5]. The Reduction of Early Mortality in HIV-Infected Adults and Children Starting Antiretroviral Therapy (REALITY) trial conducted in Zimbabwe, Kenya, Malawi, and Uganda found that enhanced prophylaxis at the time of ART initiation with ongoing cotrimoxazole prophylaxis, 12 weeks of isoniazid/pyridoxine, 5 days of azithromycin, and a single dose of albendazole was associated with a 25% reduction in early mortality among adults and children with CD4+ counts < 100 cells/mm^3^ [38]. The Reduction of Early Mortality among HIV-Infected Subjects Starting Antiretroviral Therapy (REMSTART) trial conducted in Tanzania and Zambia, in which patients at clinics were assigned to a combination strategy that included screening for cryptococcal disease coupled with 4 weeks of home visits for monitoring response and ART adherence support, resulted in a 28% decrease in mortality when compared to the standard of care [39]. For patients at high risk of loss to follow-up, structural and behavioral interventions such as accelerated ART initiation, phone messaging and texting for appointment reminders, tailored community support, and transport vouchers may be necessary to enhance retention in care [40].

## Conclusions

Over the past decade, the scale-up of HIV services has saved millions of lives, but a substantial proportion of patients continue to suffer poor health outcomes, particularly in the first year of treatment. Clinical decision tools, including risk scores, are urgently needed to promptly identify such patients and to guide them to appropriately tailored services that offer them individual benefits while at the same time contributing to the efficient use of health system resources.

## Abbreviations

ART: antiretroviral therapy
PY: person year
REALITY: Reduction of Early Mortality in HIV-Infected Adults and Children Starting Antiretroviral Therapy
REMSTART: Reduction of Early Mortality among HIV-Infected Subjects Starting Antiretroviral Therapy
TB: tuberculosis
WHO: World Health Organization

## References

[1] UNAIDS. UNAIDS Website. http://www.unaids.org/en/resources/presscentre/pressreleaseandstatementarchive/2015/july/20150714_PR_MDG6report. Geneva: UNAIDS; 2016 [Accessed 2016 May 24].

[2] UNAIDS. 90-90-90 An Ambitious treatment target to help end the AIDS epidemic. http://www.unaids.org/sites/default/files/media_asset/90-90-90_en_0.pdf. Geneva: UNAIDS, 2014.

[3] McNairy ML, El-Sadr WM. The HIV care continuum: no partial credit given. AIDS. 2012;26(14):1735–8. 10.1097/QAD.0b013e328355d67b 22614888

[4] The Global Fund. Differentiated care for HIV and Tuberculosis: A toolkit for health facilities. http://www.theglobalfund.org/documents/core/infonotes/Core_DifferentiatedCare_Toolkit_en/ Geneva: The Global Fund; 2016 [Accessed 2016 May 8].

[5] WHO. HIV Treatment and Care, What's New in Service Delivery Fact Sheet. http://apps.who.int/iris/bitstream/10665/204461/1/WHO_HIV_2015.46_eng.pdf?ua=1. Geneva: WHO; 2015 [Accessed 2015 November 20].

[6] Ellman T. Demedicalizing AIDS prevention and treatment in Africa. N Engl J Med. 2015;372(4):303–5. 10.1056/NEJMp1414730 25607425

[7] Decroo T, Koole O, Remartinez D, dos Santos N, Dezembro S, Jofrisse M, et al Four-year retention and risk factors for attrition among members of community ART groups in Tete, Mozambique. Trop Med Int Health. 2014;19(5):514–21. 10.1111/tmi.12278 24898272

[8] Tsondai P WL, Grimsrud A, Mdlalo P, Trivino A, Boulle A. High rates of retention and viral suppression in the scale-up of antiretroviral therapy adherence clubs in Cape Town, South Africa. 21st International AIDS Conference; July 18–22, 2016; Durban, South AFrica 2016. 10.7448/IAS.20.5.21649 PMC5577696 28770595

[9] Grimsrud A, Lesosky M, Kalombo C, Bekker LG, Myer L. Implementation and Operational Research: Community-Based Adherence Clubs for the Management of Stable Antiretroviral Therapy Patients in Cape Town, South Africa: A Cohort Study. J Acquir Immune Defic Syndr. 2016;71(1):e16–23. 10.1097/QAI.0000000000000863 26473798

[10] Koenig SP, Rodriguez LA, Bartholomew C, Edwards A, Carmichael TE, Barrow G, et al Long-term antiretroviral treatment outcomes in seven countries in the Caribbean. J Acquir Immune Defic Syndr. 2012;59(4):e60–71. 10.1097/QAI.0b013e318245d3c1 22240464 PMC3299899

[11] Lawn SD, Harries AD, Anglaret X, Myer L, Wood R. Early mortality among adults accessing antiretroviral treatment programmes in sub-Saharan Africa. AIDS. 2008;22(15):1897–908. 10.1097/QAD.0b013e32830007cd 18784453 PMC3816249

[12] McNairy ML, Lamb MR, Abrams EJ, Elul B, Sahabo R, Hawken MP, et al Use of a Comprehensive HIV Care Cascade for Evaluating HIV Program Performance: Findings From 4 Sub-Saharan African Countries. J Acquir Immune Defic Syndr. 2015;70(2):e44–51. 10.1097/QAI.0000000000000745 26375466 PMC5094356

[13] McNairy ML, Lamb MR, Carter RJ, Fayorsey R, Tene G, Mutabazi V, et al Retention of HIV-infected children on antiretroviral treatment in HIV care and treatment programs in Kenya, Mozambique, Rwanda, and Tanzania. J Acquir Immune Defic Syndr. 2013;62(3):e70–81. 10.1097/QAI.0b013e318278bcb0 23111575 PMC5094048

[14] Lawn SD, Harries AD. Reducing tuberculosis-associated early mortality in antiretroviral treatment programmes in sub-Saharan Africa. AIDS. 2011;25(12):1554–5; author reply 6. 10.1097/QAD.0b013e328348fb61 21747238

[15] Liechty CA, Solberg P, Were W, Ekwaru JP, Ransom RL, Weidle PJ, et al Asymptomatic serum cryptococcal antigenemia and early mortality during antiretroviral therapy in rural Uganda. Trop Med Int Health. 2007;12(8):929–35. 10.1111/j.1365-3156.2007.01874.x 17697087

[16] Fenner L, Brinkhof MW, Keiser O, Weigel R, Cornell M, Moultrie H, et al Early mortality and loss to follow-up in HIV-infected children starting antiretroviral therapy in Southern Africa. J Acquir Immune Defic Syndr. 2010;54(5):524–32. 10.1097/QAI.0b013e3181e0c4cf 20588185 PMC2925143

[17] Fox MP, Rosen S. Retention of Adult Patients on Antiretroviral Therapy in Low- and Middle-Income Countries: Systematic Review and Meta-analysis 2008–2013. J Acquir Immune Defic Syndr. 2015;69(1):98–108. 10.1097/QAI.0000000000000553 25942461 PMC4422218

[18] Machine EM, Gillespie SL, Homedes N, Selwyn BJ, Ross MW, Anabwani G, et al Lost to follow-up: failure to engage children in care in the first three months of diagnosis. AIDS Care. 2016;28(11):1402–10. 10.1080/09540121.2016.1179714 27160542 PMC5070915

[19] Wilkinson LS, Skordis-Worrall J, Ajose O, Ford N. Self-transfer and mortality amongst adults lost to follow-up in ART programmes in low- and middle-income countries: systematic review and meta-analysis. Trop Med Int Health. 2015;20(3):365–79. 10.1111/tmi.12434 25418366

[20] Lahuerta M, Wu Y, Hoffman S, Elul B, Kulkarni SG, Remien RH, et al Advanced HIV disease at entry into HIV care and initiation of antiretroviral therapy during 2006–2011: findings from four sub-saharan African countries. Clin Infect Dis. 2014;58(3):432–41. 10.1093/cid/cit724 24198226 PMC3890338

[21] Braitstein P, Songok J, Vreeman RC, Wools-Kaloustian KK, Koskei P, Walusuna L, et al "Wamepotea" (they have become lost): outcomes of HIV-positive and HIV-exposed children lost to follow-up from a large HIV treatment program in western Kenya. J Acquir Immune Defic Syndr. 2011;57(3):e40–6. 10.1097/QAI.0b013e3182167f0d 21407085 PMC3145828

[22] WHO. World Health Organization/International Society of Hypertension risk prediction chart for cardiovascular disease. http://www.who.int/cardiovascular_diseases/guidelines/Chart_predictions/en/. Geneva: WHO/ISH; 2007 [Accessed 2016 May 6].

[23] Fine MJ, Auble TE, Yealy DM, Hanusa BH, Weissfeld LA, Singer DE, et al A prediction rule to identify low-risk patients with community-acquired pneumonia. N Engl J Med. 1997;336(4):243–50. 10.1056/NEJM199701233360402 8995086

[24] Quinn J, McDermott D, Stiell I, Kohn M, Wells G. Prospective validation of the San Francisco Syncope Rule to predict patients with serious outcomes. Annals of emergency medicine. 2006;47(5):448–54. 10.1016/j.annemergmed.2005.11.019 16631985

[25] Tate JP, Justice AC, Hughes MD, Bonnet F, Reiss P, Mocroft A, et al An internationally generalizable risk index for mortality after one year of antiretroviral therapy. AIDS. 2013;27(4):563–72. 10.1097/QAD.0b013e32835b8c7f 23095314 PMC4283204

[26] Robbins GK, Johnson KL, Chang Y, Jackson KE, Sax PE, Meigs JB, et al Predicting virologic failure in an HIV clinic. Clin Infect Dis. 2010;50(5):779–86. 10.1086/650537 20121574 PMC3101804

[27] Mocroft A, Ledergerber B, Zilmer K, Kirk O, Hirschel B, Viard JP, et al Short-term clinical disease progression in HIV-1-positive patients taking combination antiretroviral therapy: the EuroSIDA risk-score. AIDS. 2007;21(14):1867–75. 10.1097/QAD.0b013e328270b877 17721094

[28] Boulle A, Schomaker M, May MT, Hogg RS, Shepherd BE, Monge S, et al Mortality in patients with HIV-1 infection starting antiretroviral therapy in South Africa, Europe, or North America: a collaborative analysis of prospective studies. PLoS Med. 2014;11(9):e1001718 10.1371/journal.pmed.1001718 25203931 PMC4159124

[29] Amuron B, Namara G, Birungi J, Nabiryo C, Levin J, Grosskurth H, et al Mortality and loss-to-follow-up during the pre-treatment period in an antiretroviral therapy programme under normal health service conditions in Uganda. BMC Public Health. 2009;9:290 10.1186/1471-2458-9-290 19671185 PMC2734853

[30] Etard J-F, Ndiaye I, Thierry-Mieg M, Guèye NFN, Guèye PM, Lanièce I, et al Mortality and causes of death in adults receiving highly active antiretroviral therapy in Senegal: a 7-year cohort study. AIDS. 2006;20(8):1181–9. 10.1097/01.aids.0000226959.87471.01 16691070

[31] Lawn SD, Myer L, Harling G, Orrell C, Bekker LG, Wood R. Determinants of mortality and nondeath losses from an antiretroviral treatment service in South Africa: implications for program evaluation. Clin Infect Dis. 2006;43(6):770–6. 10.1086/507095 16912954

[32] Koenig SP, Bernard D, Devieux JG, Atwood S, McNairy ML, Severe P, et al Trends in CD4 Count Testing, Retention in Pre-ART Care, and ART Initiation Rates over the First Decade of Expansion of HIV Services in Haiti. PLoS ONE. 2016;11(2):e0146903 10.1371/journal.pone.0146903 26901795 PMC4763018

[33] Rachlis B, Ochieng D, Geng E, Rotich E, Ochieng V, Maritim B, et al Implementation and operational research: evaluating outcomes of patients lost to follow-up in a large comprehensive care treatment program in western Kenya. J Acquir Immune Defic Syndr. 2015;68(4):e46–55. 10.1097/QAI.0000000000000492 25692336 PMC4348019

[34] Brennan AT, Maskew M, Sanne I, Fox MP. The importance of clinic attendance in the first six months on antiretroviral treatment: a retrospective analysis at a large public sector HIV clinic in South Africa. J Int AIDS Soc. 2010;13:49 10.1186/1758-2652-13-49 21134297 PMC3012655

[35] Wijeysundera DN. Predicting outcomes: Is there utility in risk scores? Canadian Journal of Anesthesia/Journal canadien d'anesthésie. 2016;63(2):148–58. 10.1007/s12630-015-0537-2 26670801

[36] Reilly BM, Evans AT. Translating clinical research into clinical practice: impact of using prediction rules to make decisions. Ann Intern Med. 2006;144(3):201–9. 16461965 10.7326/0003-4819-144-3-200602070-00009

[37] Toll BA, Katulak NA, Williams-Piehota P, O'Malley S. Validation of a scale for the assessment of food cravings among smokers. Appetite. 2008;50(1):25–32. 10.1016/j.appet.2007.05.001 17574300 PMC2386855

[38] Hakim VM J., Szubert A.J., Siika A., Mallewa J., Agutu C., Pett S.L., Bwakura-Dangarembizi M., Lugemwa A., Kaunda S., Karoney M., Maitland K., Griffiths A., Kityo C., Mugyenyi P., Prendergast A.J., Walker A.S., Gibb D.M., REALITY Trial Team. Enhanced infection prophylaxis reduces mortality in severely immunosuppressed HIV-infected adults and older children initiating antiretroviral therapy in Kenya, Malawi, Uganda and Zimbabwe: the REALITY trial. AIDS 2016; Durban, South Africa2016.

[39] Mfinanga S, Chanda D, Kivuyo SL, Guinness L, Bottomley C, Simms V, et al Cryptococcal meningitis screening and community-based early adherence support in people with advanced HIV infection starting antiretroviral therapy in Tanzania and Zambia: an open-label, randomised controlled trial. Lancet. 2015;385(9983):2173–82. 10.1016/S0140-6736(15)60164-7 25765698

[40] McNairy ML, Gachuhi AB, Lamb MR, Nuwagaba-Biribonwoha H, Burke S, Ehrenkranz P, et al The Link4Health study to evaluate the effectiveness of a combination intervention strategy for linkage to and retention in HIV care in Swaziland: protocol for a cluster randomized trial. Implementation science: IS. 2015;10:101 10.1186/s13012-015-0291-4 26189154 PMC4506770

